# Interstitial Cystitis-Associated Urinary Metabolites Identified by Mass-Spectrometry Based Metabolomics Analysis

**DOI:** 10.1038/srep39227

**Published:** 2016-12-15

**Authors:** Tobias Kind, Eunho Cho, Taeeun D. Park, Nan Deng, Zhenqiu Liu, Tack Lee, Oliver Fiehn, Jayoung Kim

**Affiliations:** 1 West Coast Metabolomics Center, University of California, Davis, Davis, CA, USA; 2 University of California Los Angeles, CA, USA; 3 University of California, Berkerly, CA, USA; 4 Samuel Oschin Comprehensive Cancer Institute, Cedars-Sinai Medical Center, Los Angeles, CA, USA; 5 Department of Urology, Inha University College of Medicine, Incheon, South Korea; 6 King Abdulaziz University, Jeddah, Saudi Arabia; 7 Departments of Surgery and Biomedical Sciences, Cedars-Sinai Medical Center, Los Angeles, CA, USA.; 8 Department of Medicine, University of California Los Angeles, Los Angeles, CA, USA.

## Abstract

This study on interstitial cystitis (IC) aims to identify a unique urine metabolomic profile associated with IC, which can be defined as an unpleasant sensation including pain and discomfort related to the urinary bladder, without infection or other identifiable causes. Although the burden of IC on the American public is immense in both human and financial terms, there is no clear diagnostic test for IC, but rather it is a disease of exclusion. Very little is known about the clinically useful urinary biomarkers of IC, which are desperately needed. Untargeted comprehensive metabolomic profiling was performed using gas-chromatography/mass-spectrometry to compare urine specimens of IC patients or health donors. The study profiled 200 known and 290 unknown metabolites. The majority of the thirty significantly changed metabolites before false discovery rate correction were unknown compounds. Partial least square discriminant analysis clearly separated IC patients from controls. The high number of unknown compounds hinders useful biological interpretation of such predictive models. Given that urine analyses have great potential to be adapted in clinical practice, research has to be focused on the identification of unknown compounds to uncover important clues about underlying disease mechanisms.

More than 3–8 million women and 1–4 million men are diagnosed with Interstitial Cystitis (IC), also known as Painful Bladder Syndrome, in the US annually^1^. IC impacts health-related qualities of life immensely, and in some instances can be more debilitating than end-stage renal disease^2^,^3^. In spite of an increase in the number of diagnosed cases, objective diagnostic criteria are not consistently applied in general practice^4^. Some lower urinary tract symptoms, such as overactive bladder (OAB), have symptoms in common with IC, further complicating the diagnosis. Diagnosis of the disease has been dependent on clinical parameters (e.g. pain, urgency, and frequency) due to the lack of proper conventional markers (e.g. PSA for prostate cancer diagnosis)^3^,^5^. Diagnostic tests include urinalysis, urine culture, cystoscopy, bladder biopsy and hydrodistention of the bladder. Nonetheless, we still lack definite criteria for the disease. Estimates of the prevalence and natural history of IC still fluctuate widely because of different diagnostic standards, populations evaluated, and challenges inherent in following patients over time^6^. Thus, the identification of sensitive and non-invasive biomarkers has the potential to greatly improve the accuracy of an IC diagnosis. However, our current understanding of mechanisms involving pelvic pain is also unclear and fragmented.

Urinary metabolites represent a signature of a subject’s metabolic state and may convey critical information about the pathophysiology of disease. This may be especially true for pelvic disorders because urine is the body fluid most proximal to the urinary tract. Because metabolites vary in size, chemistry and physicochemical properties, a single platform has only a limited capacity to interrogate the entire metabolome in a given body fluid. Use of more than one platform spanning different technologies is the preferred means of performing comprehensive metabolome analyses. Urine excretions represent a snapshot of many metabolic endpoints including those from food, drugs, nutrients and bacterial transformations. This renders urine analysis very challenging due to the complexity, sources and numbers of metabolites.

In this study, we performed gas-chromatography time of flight mass spectrometry (MS)-based metabolomics analysis. Our goal here was to increase coverage of known metabolites that may play a role in IC and to gain new insight into disease mechanisms. Previous global metabolomics profiling of urine from IC patients suggests that a urinary metabolic signature for IC can be detected using platforms such as Nuclear Magnetic Resonance (NMR) and Liquid chromatography–mass spectrometry (LC-MS). The experimental results from this paper suggest that candidate metabolites were found to be associated with IC, and that the IC metabolic signature can be identified in patient urine. Using multiparametric models such partial least squares discriminant analysis IC metabolic signature can stratify patients from control subjects.

## Results

### Characteristics of the study subjects

A clinical diagnosis of IC was made by two independent urologists, according to NIDDK criteria (e.g. frequency, urgency, bladder pain, discomfort and the presence of glomerulations during cystoscopic hydrodistention), before any treatment or medication was given. Only subjects of >2 month “free of treatment or medication” were included. In total, we enrolled 63 female subjects (42 IC patients and 21 normal controls) with a mean age of 51. Given that most of patients (over 80%) are women, we recruited only female patients for this particular study to seek potential sex-specific urine biomarkers for female IC patients. Population-based, age-matched controls were recruited from one clinic using the same standard operating procedures (SOPs) during the same research period (2010–2013).

### GC-TOF MS analysis of urine specimens from IC patients and controls

We investigated the metabolite profile of the individual urine samples using GC-TOF mass spectrometry. Our analysis and data requisition resulted in a total of 490 metabolites detected (200 known and 290 unknown metabolites).

**Figure 1:**
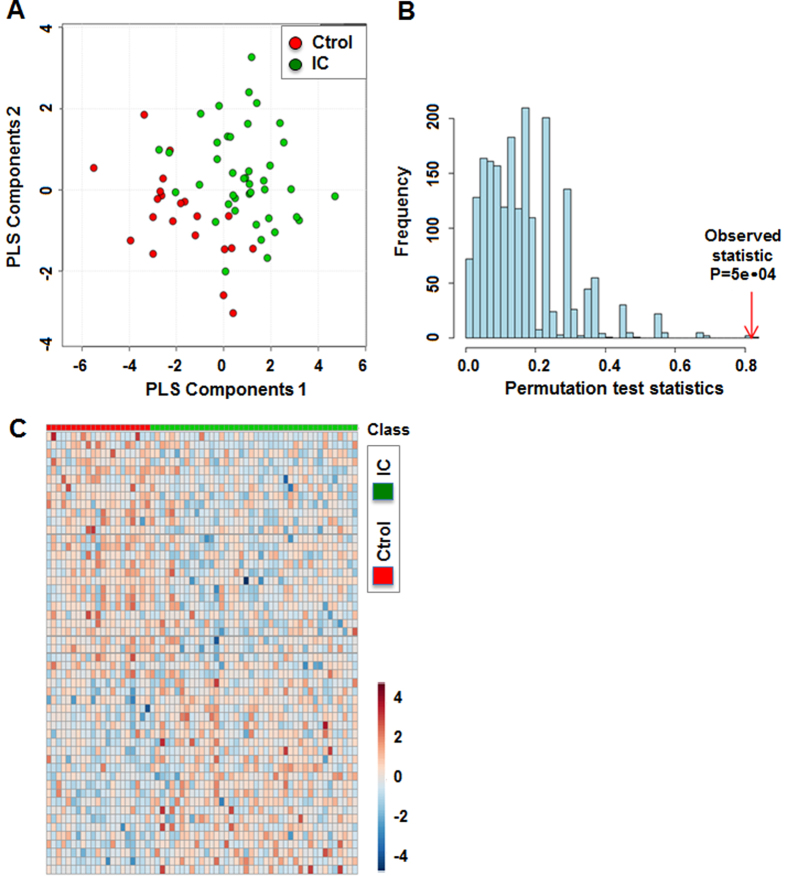
Differentiation of IC patients and healthy control groups using multivariate analysis. (**A**) Partial least square-discriminant analysis (PLS-DA) score plot of the IC and control groups. PLS-DA plot showed a clear separation of metabolites between patients and matched control subjects. Red: control samples; Green: IC patient samples. The model was established using three principal components. (**B**) For model evaluation, the class prediction results based on cross model validation predictions of the original labeling compared to the permuted data assessed using the separation distance. Histogram shows distribution of separation distance based on permutated data. Red arrow indicates observed statistic (P = 5e-04). (**C**) A heatmap of 52 differentially expressed metabolites in IC and control groups. Among 490 detected metabolites in total, 52 metabolites, including both annotated and unannotated metabolites, were significantly altered in IC patients compared to controls (FC > 1.20 or FC < 0.83 and P < 0.1).

Data were autoscaled and mean-centered. The scores plot for partial least squares (PLS) components showed differentiation of the IC samples from controls with good separation and dispersion (Fig. 1A). We assessed the accuracy of our predictive model using the leave-one-out cross-validation method as well as the randomized permutation (Fig. 1B). The observed statistic of this analysis using MetaboAnalyst 3.0 software^1^ was significant at p = 0.005, suggesting that the model significantly differentiate patients from healthy controls. A heat map also showed the distinct expression patterns of metabolites between IC and controls (Fig. 1C). These metabolites are responsible for the significant difference between IC and controls with fold change either greater than 1.20 or less than 0.83 and p-value less than 0.1.

### Identification of differentially expressed metabolites in urine of IC patients

**Figure 2:**
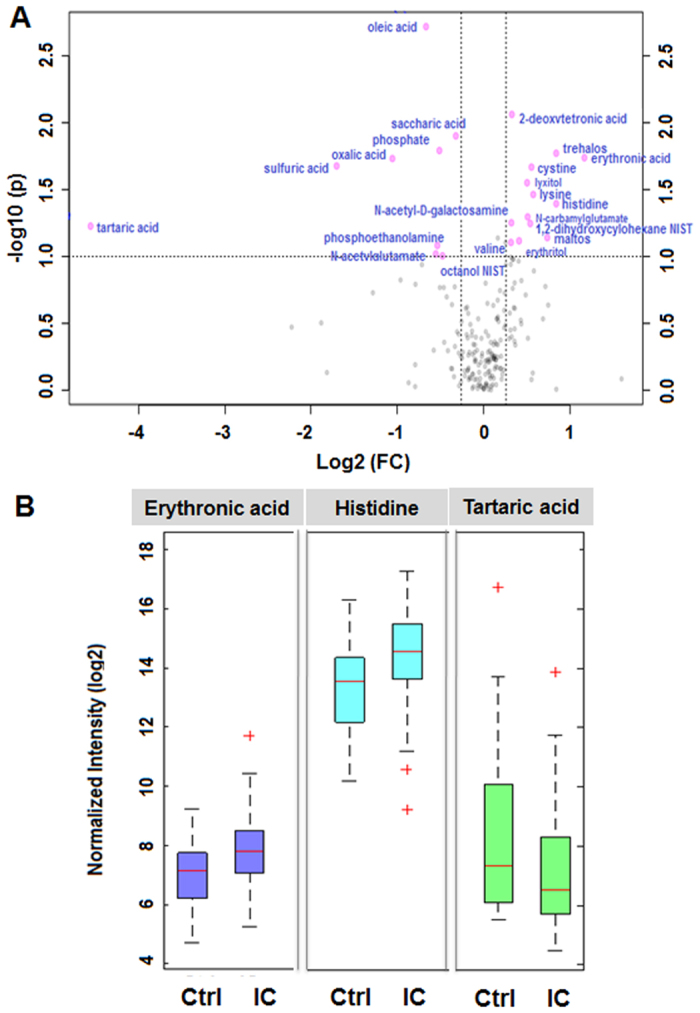
A volcano plot showing differentially expressed metabolites in IC patients. (**A**) 22 annotated metabolites were significantly altered in IC patients compared to controls (FC > 1.20 or FC < 0.83 and P < 0.1). The red dots represent metabolites above the threshold. The further the metabolite’s position away from the (0, 0), the more significant the metabolite is. (**B**) A boxplot showing up-regulated and down-regulated metabolites that could be used to differentiate IC patients from normal subjects. The candidate metabolites, erythronic acid and histidine, were significantly increased in IC patients compared to that in controls, while tartaric acid was significantly decreased. All metabolites show statistical significance with p-value < 0.1.

Given 490 detected metabolites, we investigated 52 differentially expressed metabolites, including both annotated and unannotated metabolites. In the volcano plot (Fig. 2A), annotated metabolites are presented as log2 fold change against the –log10 (p) of the differential expression between IC patients and healthy controls. 22 annotated differentially expressed metabolites above the threshold (FC > 1.20 or FC < 0.83, and P < 0.1) are marked and presented. Erythronic acid and histidine, were the most upregulated metabolites in the IC patient group compared to that in control, while tartaric acid were the most downregulated as shown in Fig. 2B and Table 1.

### Network modeling derived from IC-associated metabolites

**Figure 3:**
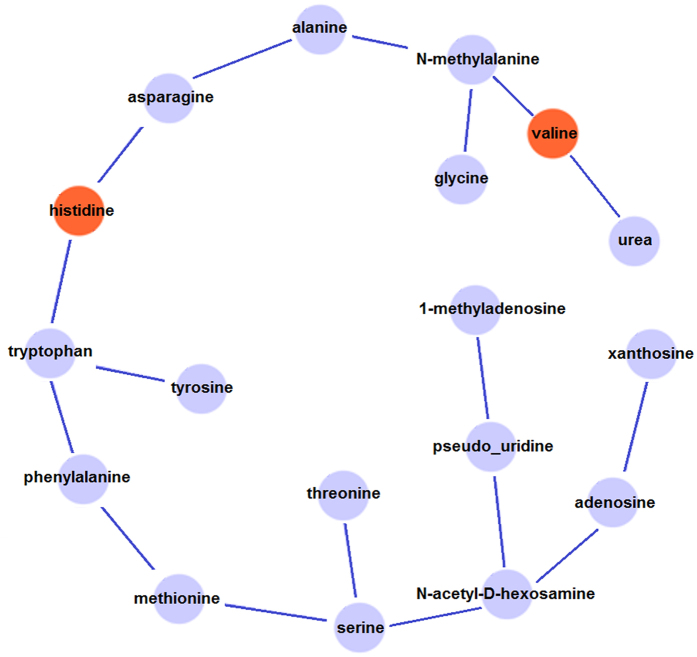
Network modeling derived from IC-associated metabolites. Histidine associated differential module (subnetwork) is shown, where the red nodes indicate upregulated metabolites and light blue nodes represents non-differentiated metabolites.

We performed analysis the histidine-associated differential module (subnetwork) using multilevel local graphical model^7^ (Fig. 3). The differential network represents the changes of correlation structure in IC when compared to the background network. Levels of two metabolites, valine and histidine (in red circle), are increased in IC. The interactions (correlations) among metabolites indicate that those metabolites may biologically function together. Generally, the variations of interactions among metabolites under different clinical conditions are associated with IC status. Sparse local graphical model^8^ is used to construct both common and differential metabolite networks simultaneously. Treating each metabolite, in turn, as the response variable and the remaining annotated metabolites as predictors, and running the sparse regression built the network. In such an approach, for each metabolite *x*_*i*_, the regression model is defined as


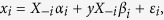


where *X*_−*i*_ are the metabolite expression values except for metabolite *x*_*i*_, and *y* (1/0) represents IC (1) or control (0). The common and differential networks are formed by collecting all of the *α*_*i*_*s* and *β*_*i*_*s*, respectively. Parameters (*α*_*i*_) determine the direct correlations between metabolite *x*_*i*_ and the remaining metabolites, and *α*_*ij*_ ≠ 0 indicate there is a partial correlation (edge) between metabolites *x*_*i*_ and *x*_*j*_, giving the remaining metabolites. Moreover, *β*_*i*_ measure *y* dependent associations and differential correlations across different clinical condition. Parameter *β*_*ij*_ ≠ 0 indicates that there is a differential interaction between metabolites *x*_*i*_ and *x*_*j*_ in IC and control.

**Figure 4:**
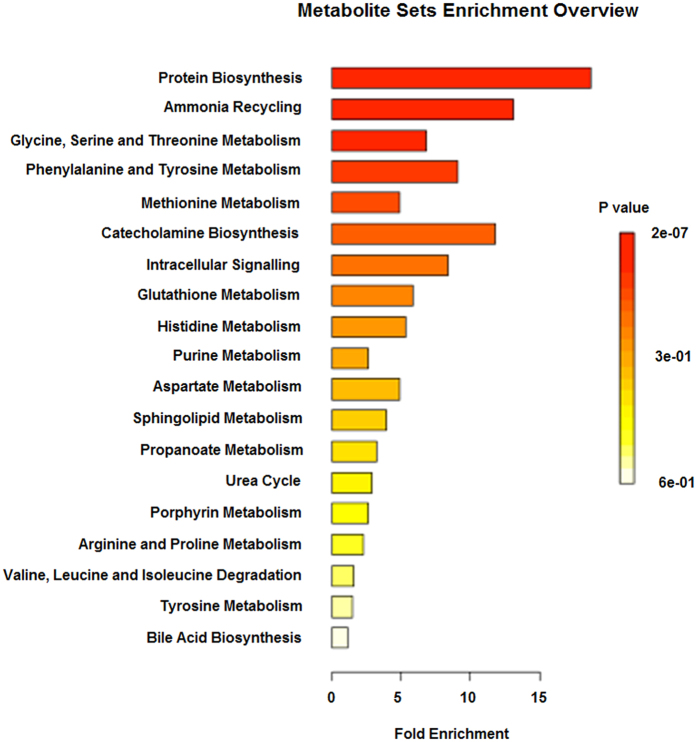
Differential network in IC is identified with multilevel local graphical model^7^. The differential network represents the changes of correlation structure in IC when compared to the background network. Two metabolites (in red) are also upregulated in IC. The interactions (correlations) among metabolites indicate those metabolites may function together biologically. Metabolite Set Enrichment Analysis (MESA) with the 18 metabolites shows that those metabolites are highly enriched in Protein Biosynthesis and Ammonia Recycling with the FDR of 0.0000136 and 0.00557, respectively. The following is the result of MSEA.

Cytoscape (www.cytoscape.org/) was used for differential network visualization and subnetwork identification. The proposed approach identified the IC associated differential network efficiently (Fig. 3). For further understanding on our metabolite signature, software MetaboAnalyst was used for functional enrichment analysis. Metabolite enrichment analysis allows us to study the corresponding biological pathways of IC with metabolites on the differential network. We performed Metabolite Set Enrichment Analysis (MSEA) with the 18 metabolites, which were derived from data in Fig. 3. We found that those 18 metabolites are highly enriched in Protein Biosynthesis and Ammonia Recycling with the FDR of 0.0000136 and 0.00557, respectively (Fig. 4).

## Discussion

In this study we profiled 490 metabolites in human urine specimens for IC diagnosis using GC-TOF MS. Metabolites including histidine, erythronic acid, and tartaric acid were found to have the highest fold-changes. Power analysis and false discovery rate correction (FDR, Benjamini-Hochberg) suggests that the study sample size has to be increased to validate any findings. The present report has provided evidence that metabolic fingerprints can predict IC patients using multiparametric models such as PLS-DA, however it remains to be determined whether these metabolites might have biological and mechanistic meanings. Especially the large number of unknown compounds is challenging (59% in this study), because without structural annotation, unnknown metabolites can only be partly assigned to larger biochemical modules through mass spectral similarity analysis. Some unknowns may even ultimately prove to be chemical contaminants and should be excluded from multiparametric models. One solution to increase mass spectral library coverage is to use quantum chemical simulations predict electron ionization mass spectra^9^ or to utilize novel machine learning methods to improve compound identification^10^. This can also include novel metabolic compounds that can be expected to exist from known metabolic transformations^11^.

Histidine, one of essential amino acids in humans, is a known precursor of the neurotransmitter histamine. Increased histidine level leads to increase of histamine level in blood, brain and possibly bladder, suggesting the possibility that histidine may have many other possible functions affecting human bladder sensory system. Previous work using IC rat model demonstrated that overexpression of monocyte chemo-attractant protein-1 (MCP-1) in bladder tissues contributes histamine production and IC^12^. More recently, findings from animal model suggest that mast cell-derived histamine mediates IC-associated pain. Authors showed that histamine receptors 1 and 2 modulate pelvic pain and antihistamines attenuate bladder pain in their animal model. We believe the simplest explanation for this finding is that an increased secretion of histamine and histidine (precursor of histamine) may be associated with IC symptoms mediated by mast cells infiltrated in bladder. Other candidate metabolites from our study are summarized in Table 1.

Previous studies have suggested a series of IC biomarker candidates, including antiproliferative factor^13^, phenylacetylglutamine^14^, interleukin-6, histamines^15^, nerve growth factor *et al*. Our laboratory also found tyramine and 2-oxoglutarate as urinary biomarkers for IC diagnosis^16^. More recently, the Multidisciplinary Approach to the Study of Chronic Pelvic Pain (MAPP) Research Network identified Etio-S (etiocholan-3α-ol-17-one sulfate) to discriminate IC patients from healthy controls^17^. This urinary sulfometabolome profiling study was performed using Liquid Chromatography–Mass Spectrometry (LC–MS) in female subjects who had high symptom scores as well as high pelvic pain/pressure/discomfort scores.

Metabolic fingerprints shown in a heatmap (Fig. 1C) consist of 22 annotated metabolites among 52 metabolites shown in a heatmap (Fig. 1C) including histidine, valine, tartaric acid, and erythronic acid *et al*. These metabolites are listed in Table 1. This metabolic fingerprint might be applicable to segregate IC patients from healthy controls in the clinical setting, although it is out of scope of this study.

Urine analysis is certainly challenging due to its high biological variance, because urine is a sink for all water soluble metabolites coming from food sources, the microbiome, drugs, chemicals and generally the exposome. However urine can be collected non-invasively, across all age ranges and in large quantities compared to blood, it is also an excellent matrix for personalized clinical profiles.

For robust statistical analysis many confounding factors such as age, race, geographical location or food intake have to be considered. Subject meta-data may be collected through questionnaires at time of sample collection in the clinic, but it can also be assessed through thorough chemical profiling analyses, called exposome screening (e.g. for pharmaceutical agents or food biomarkers). For example the compound 2-furoylglycine can be used to diagnose fatty acid beta-oxidation disorders, but is also found in food prepared by strong heating (http://www.hmdb.ca/metabolites/HMDB00439). Cotinine is a known marker for exposure to cigarette smoke, and other metabolites are known food markers such as caffeine and theobromine for coffee consumption. Such markers can be easily collected along with metabolomic analyses and could be used to stratify patient cohorts or to adjust for exposure parameters during data analysis.

Urine metabolite levels are currently collected from published reports^18^. However individual urinary metabolite levels are currently not collected in large databases. Therefore it is difficult to determine minimum, mean, maximum levels of specific metabolites or to perform correlations to dietary intake, which would affect the validity of certain biomarkers. Here efforts have to be undertaken to collect such profiles, similar to personalized efforts that will sequence individual humans or collect individual metabolic profiles from blood.

In summary, our GC-TOF MS analysis suggested a number of metabolite candidates associated with IC. Large cohorts have to be utilized to validate predictive biomarkers or models. This method may provide novel opportunities for better diagnosis and clinical management of IC, particularly in a non-invasive manner. A major clinical challenge remains the early diagnosis of IC. Given that these current findings from this study, although it is out of scope of this study, however we will aim to test whether abnormal metabolism is a key hallmark of IC as a next step. Our metabolic biomarker panel provides the prospect for assisting predictive factor to determine severity of urinary symptoms and pain/discomfort of IC patients.

## Materials and Methods

### Ethics statement

The Ethics Committee of Inha University Hospital in South Korea approved this study. The Institutional Review Board of Inha University Hospital approved collection, curation and analysis of all samples. All subjects participated in this study provided written informed consent, and all experiments were performed in accordance with relevant guidelines and regulations.

### Subjects and urine specimen collection

IC patients and healthy control subjects were diagnosed and recruited from an outpatient urology clinic at Inha University Hospital. Work-up included symptom assessment, cystoscopic evaluation, physical examination, urodynamics, and/or urine culture. Patients with a history of other diseases (such as any types of cancer, inflammation, or diabetes, etc.) were excluded. All subjects were of Asian female descent resident in South Korea. To avoid possible contamination with vaginal or urethral cells, first morning urine specimens were obtained using clean catch methods in a sterile environment. The de-identified specimens were sent to clinical laboratory and were centrifuged to remove cell debris. Supernatants were processed into individual aliquots of 1 ml/tube, before storage at −80 °C until further analysis.

### GC TOF-MS analysis of urine

The gas-chromatography/mass-spectrometry (GC-MS) analysis was performed^19^,^20^. Normally, 10 ul of urine are dissolved in 1 ml −20 °C cooled acetonitrile, isopropanol and water (3:3:2 v/v) mixture at pH 7. In this case the urine volume was adjusted between 2 and 10 ul to externally measured creatinine levels using a linear calibration curve. Then the solution was vortexed at 4 °C for 5 minutes in 1.5 ml Eppendorf tubes. Samples were centrifuged for 2 min at 14,000 rcf and 500 ul were aliquoted. The aliquot was the evaporated in a Labconco Centrivap cold trap to complete dryness. The methoximation step was performed with 10 ul of a solution of 40 mg/ml O-methylhydroxylamine hydrochloride (CAS: [593-56-6]; Formula CH5NO.HCl) and 90 minutes shaking at 30 °C. Then 90 ul of N-methyl-N-trimethylsilyltrifluoroacetamide (MSTFA) was added and shaken at 37 °C for 30 min. Then a mix of 1 ul fatty acid methyl esters (FAME) retention time markers was added. The mixture was transferred to amber crimp autosampler vials. Measurements were performed on a Leco Pegasus IV TOF coupled to an Agilent 6890GC with Agilent 6890 split/splitless injector. The column was a Restek RTX-5Sil MS (95% dimethyl/5% diphenyl polysiloxane) with 30 m length, 0.25 mm i.d. and 0.25 um film thickness with 10 m guard column. Injection volume was 1 ul at 250 °C. The GC parameters were set to 1 ml/min constant flow Helium and an oven ramp of 50 °C (1 min hold) to 330 °C at 20 °C/min, 5 min hold before cool-down. The transfer line temperature was 280 °C and spectra were recorded in electron ionization mode at 70 eV with a filament temperature of 250 °C TOF and scan range of 85–500 u. All the raw data was deposited to the Metabolomics Workbench repository under study ID ST000381.

### Annotation and ID of compounds

The peak and compounds detection or deconvolution was performed with the Leco ChromaTOF software. Spectra were matched against the FiehnLib mass spectral and retention index library^20^. Post-curation and peak replacements were performed with the in-house developed BinBase software and the sample matrix with all known and unknown compounds exported to a Microsoft EXCEL sheet. A total of 490 compounds were detected. 200 compounds were annotated as known compounds by retention index and mass spectral matching and 290 compounds remain unknown.

### Data processing

We excluded one subject from the IC patient group and three subjects from controls because their spectra were outliers based on PCA analysis. To identify potential metabolites as marker candidates that can discriminate IC patients from healthy subjects, we applied the following steps. Data was normalized and the t-test was applied on the log2 of the processed data. The Student’s t-test was performed to extract significant metabolites from the normalized GC-MS data. 30 metabolites had levels of *p*-value threshold <0.05. Twelve of these were known metabolites, the remainder unknown metabolites. After false positive correction (FDR) using Benjamini–Hochberg procedure none of the p-values remained significant on the chosen level of 0.05.

The volcano plot shows the fold change and the significance of each annotated metabolite. The significant metabolites were selected by volcano plot with fold change threshold >1.20 (or <0.83) and t-tests *p*-value threshold <0.1. Second, the resultant profiles, which contain profiles of 22 annotated differentially expressed metabolites, were imported into MetaboAnalyst version 3.0^1^. Log transformation and mean-centered with auto scaling were performed prior to multivariate statistical analysis. Partial least square discriminant analysis (PLS-DA) was performed, and model evaluation with permutation strategy was carried out according to a published protocol^21^.

## Additional Information

**How to cite this article:** Kind, T. *et al*. Interstitial Cystitis-Associated Urinary Metabolites Identified by Mass-Spectrometry Based Metabolomics Analysis. *Sci. Rep.* **6**, 39227; doi: 10.1038/srep39227 (2016).

**Publisher's note:** Springer Nature remains neutral with regard to jurisdictional claims in published maps and institutional affiliations.

## Figures and Tables

**Table 1:** A list of metabolites differentially expressed in IC, compared to controls (p-level = 0.005, FDR (Benjamini Hochberg).

Num	Name	Fold-change	p-value	FDR
1	Unknown BB_31554	2.56	0.000132	0.064576
2	Unknown BB_34163	0.55	0.000514	0.12586
3	oleic acid	0.63	0.001933	0.315675
4	2-deoxytetronic acid	1.26	0.008732	0.571396
5	Unknown BB_17651	0.66	0.009136	0.571396
6	saccharic acid	0.80	0.012642	0.571396
7	Unknown BB_17140	1.44	0.015588	0.571396
8	phosphate	0.70	0.016252	0.571396
9	trehalose	1.79	0.017026	0.571396
10	Unknown BB_5900	0.81	0.017487	0.571396
11	erythronic acid	2.25	0.018393	0.571396
12	Unknown BB_109809	0.56	0.018576	0.571396
13	oxalic acid	0.48	0.018665	0.571396
14	Unknown BB_34027	0.44	0.019904	0.571396
15	Unknown BB_1704	0.63	0.020865	0.571396
16	sulfuric acid	0.31	0.021197	0.571396
17	Unknown BB_23635	0.69	0.02138	0.571396
18	cystine	1.47	0.021607	0.571396
19	Unknown BB_3029	0.70	0.022156	0.571396
20	Unknown BB_12330	2.19	0.02596	0.614149
21	Unknown BB_31549	0.37	0.026702	0.614149
22	lyxitol	1.42	0.028288	0.614149
23	Unknown BB_31756	1.74	0.028827	0.614149
24	lysine	1.49	0.034624	0.706901
25	histidine	1.79	0.040576	0.743323
26	Unknown BB_31359	0.81	0.043988	0.743323
27	Unknown BB_5121	0.64	0.045462	0.743323
28	Unknown BB_100869	1.58	0.046685	0.743323
29	Unknown BB_3294	1.37	0.046907	0.743323
30	Unknown BB_31764	1.33	0.048566	0.743323
